# Unexpected discovery of *Clonorchis sinensis* by common bile duct exploration

**DOI:** 10.1002/ccr3.5434

**Published:** 2022-02-23

**Authors:** Ping Shao, Qing Chen

**Affiliations:** ^1^ Department of General Surgery Cheng Fei Hospital Chengdu China

**Keywords:** choledocholithiasis, *Clonorchis sinensis*

## Abstract

Coexisting choledocholithiasis and *Clonorchis sinensis* infection are relatively rare. We report a 39‐year‐old patient with choledocholithiasis who inadvertently found *Clonorchis sinensis* during common bile duct exploration. Our experience suggests that if preoperative imaging reveals nonspecific changes associated with choledocholithiasis, the possibility of biliary parasite infection should be suspected.

## Graphical abstract

The preoperative imaging showing a blurred lower common bile duct is unlike the dilation caused by conventional choledocholithiasis, the possibility of biliary parasite infection should be suspected.

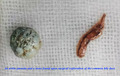

## 1 CASE DESCRIPTION

**FIGURE 1:**
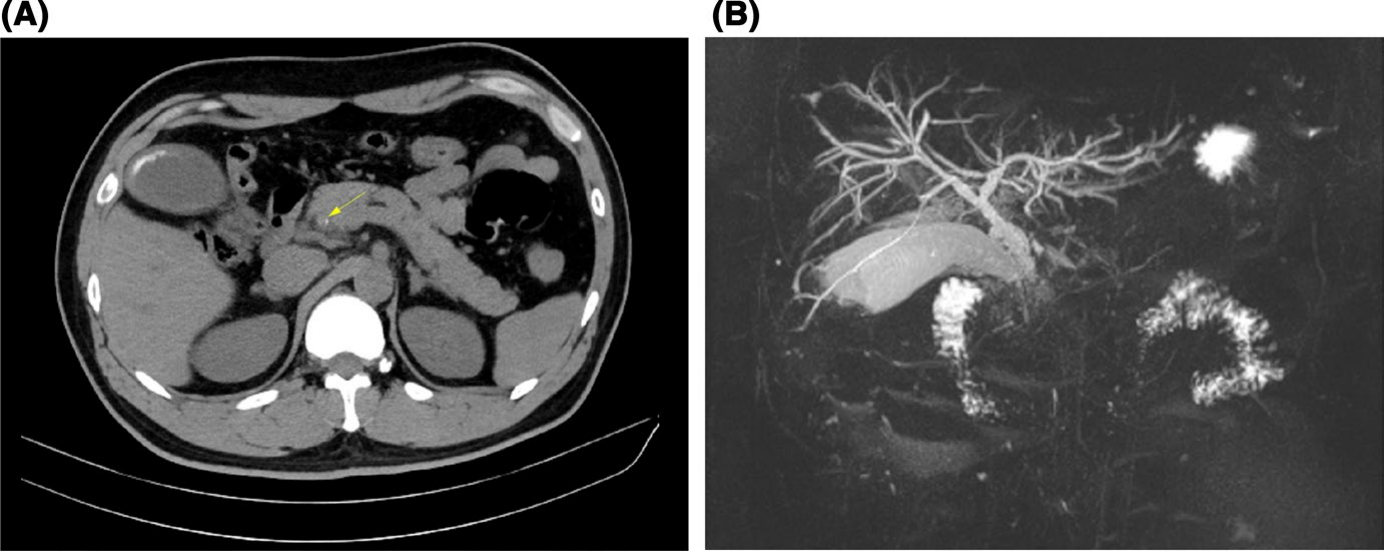
Preoperative imaging. (A) Computed tomography of the gallbladder and common bile duct stones (arrow); (B) Magnetic resonance cholangiopancreatography showing slight dilation of the intrahepatic bile duct and the unclear lower part of the common bile duct

**FIGURE 2:**
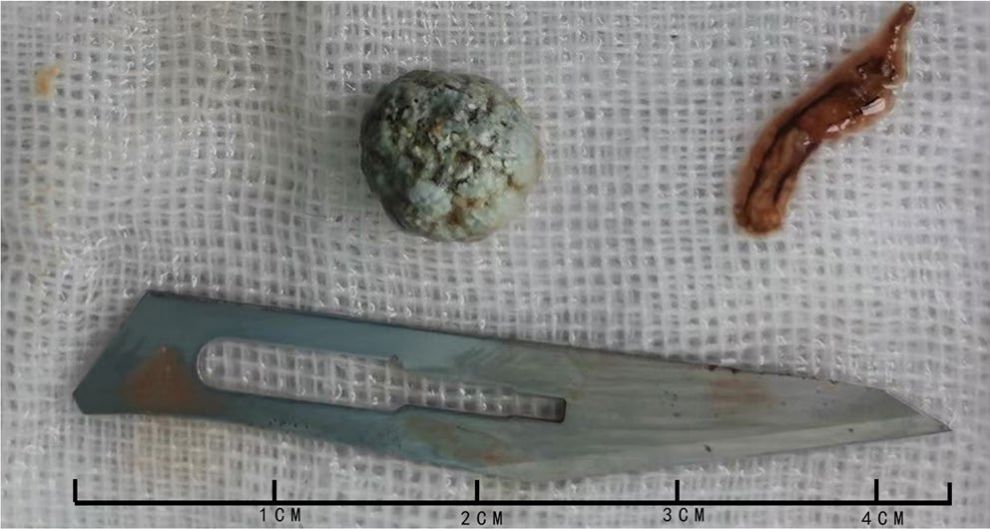
Active parasite and a stone found upon surgical exploration of the common bile duct

**FIGURE 3:**
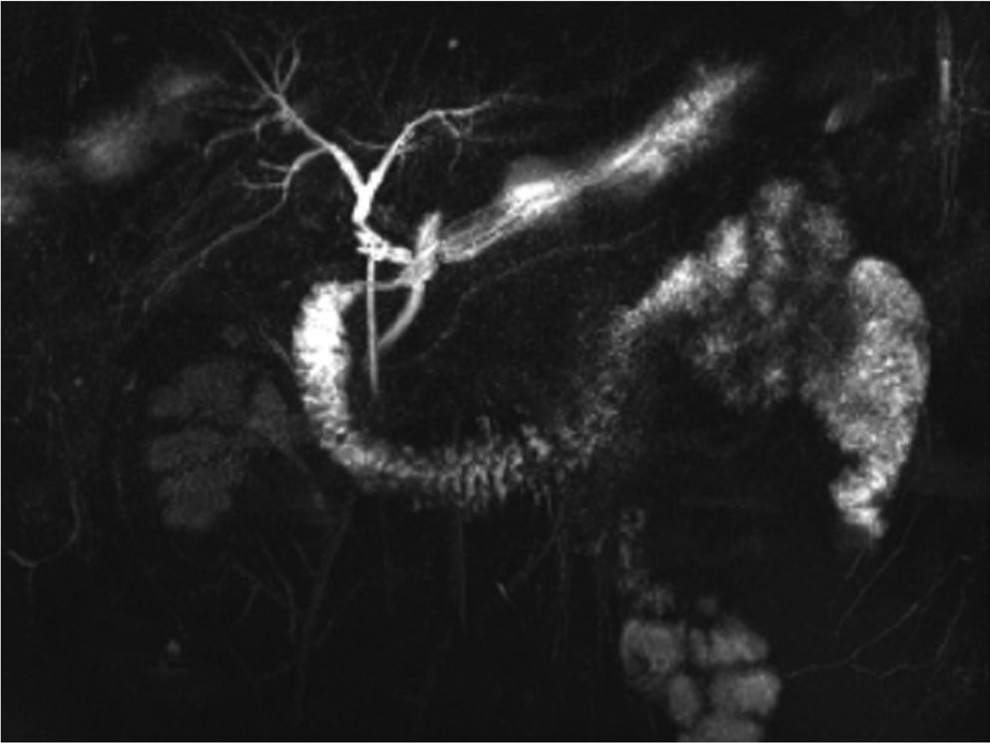
No obvious abnormalities in the bile ducts were visible by magnetic resonance cholangiopancreatography at 30 days after surgery

A 39‐year‐old man was admitted to our department with complaint of right upper quadrant abdominal pain of 5 hours duration. Increasing transaminase and bilirubin levels were the major laboratory findings. Preoperative imaging examinations are shown in Figure 1. Subsequently, laparoscopic cholecystectomy and common bile duct exploration were performed, and a T‐tube was placed for bile drainage. An active parasite (*Clonorchis* sinensis) was found during surgery (Figure 2). After surgery, the patient was given praziquantel (80 mg/kg/day for 3 days) for deworming. In the days following surgery, adult worms were not observed in the bile duct drainage. Liver function gradually recovered after surgery. No obvious bile duct abnormalities were seen (Figure 3), and the T‐tube was removed 30 days after surgery. The patient was followed for 6 months and reported experiencing no discomfort.

Previous study^1^ suggested that *Clonorchis sinensis* might cause gallstones by excretory‐secretory products or eggs. The stone found during our operation was not common cholesterol or bile pigment stone, and we considered that it might be related to *Clonorchis sinensis* infection. The preoperative imaging showing a blurred lower common bile duct is unlike the dilation caused by conventional choledocholithiasis,^2^ the possibility of biliary parasite infection should be suspected.

## CONFLICT OF INTEREST

There are no conflict of interest to declare.

## AUTHOR CONTRIBUTIONS

PS performed the data analyses and wrote the manuscript. QC designed the study and drafted the initial manuscript.

## ETHICAL APPROVAL

We confirm that the present study conforms to the ethical standards and guidelines of the journal.

## CONSENT

The written informed consent was obtained from the patient for publication of this manuscript.

## Data Availability

Data sharing is not applicable to this article as no new data were created or analyzed in this study.
